# Factors associated with compliance and non-compliance by physicians in a large-scale randomized clinical trial

**DOI:** 10.1186/1745-6215-7-26

**Published:** 2006-08-21

**Authors:** Koji Oba, Satoshi Morita, Mahbubur Rahman, Junichi Sakamoto

**Affiliations:** 1 Department of Epidemiological & Clinical Research Information Management, Kyoto University Graduate School of Medicine, Kyoto, Japan; 2 Department of Epidemiology and Health Care Research, Kyoto University Graduate School of Medicine, Kyoto, Japan; 3 Epidemiology Research Center Marshfield Clinic Research Foundation, Wisconsin, USA; 4 Young Leaders' Program of Medical Administrations and Office of International Affairs, Nagoya University Graduate School of Medicine, Nagoya, Japan

## Abstract

**Background:**

In order to minimize the amount of incomplete follow-up data, reducing the non-compliance of participating physicians is one of the key issues for the data coordinating center in a multi-center trial. Identifying the physicians' non-compliance in advance is considered to be an important strategy for more efficient conduct of trials. In this study, we identified physicians' characteristics and factors associated with the need for individual visits to institutions to collect data or to complete information during two years of follow-up in a large Japanese investigator-initiated trial related to cardiovascular disease.

**Methods:**

We categorized the physicians into two groups, "complier" and "non-complier". Odds ratios and corresponding 95% confidence intervals were calculated for 11 factors related to the characteristics of and compliance by physicians. Multiple logistic regression analysis was also performed. In addition, we evaluated the incremental cost for obtaining additional information of the non-compliant physicians.

**Results:**

Three factors were identified in multiple logistic regression analysis as being significantly associated with compliance status: 1) prior participation in clinical trials (OR = 0.40 95%CI = 0.21–0.74); 2) physician opinion that the support system for case registration and follow-up was well organized (OR = 0.41 95%CI = 0.22–0.75); and 3) number of patients recruited (OR = 2.25 95%CI = 1.01–5.02). The actual incremental cost was about US $112,000 (14.4% of total routine follow-up costs) for the non-compliant physicians during the 2 years, or about US $570 per patient.

**Conclusion:**

Investigator-initiated clinical trials have recently attracted great interest, but they often suffer from insufficient funding. If trial networks are to be well organized, it is important that trials are conducted more efficiently. We believe that our findings will be useful for reducing the additional burden associated with incomplete follow-up data and data lost to follow-up when planning future trials.

## Background

Limiting the number of patients lost to follow-up is essential for the successful completion of randomized controlled trials (RCT). In a multi-center trial, the data coordinating center is mainly responsible for locating patients who are lost during the treatment and follow-up, and persuading them to rejoin the trial [1]. However, it is usually difficult for the coordinating center staff to contact the patients directly, especially if the number of centers is large. Therefore, the coordinating center takes a variety of approaches to the participating physicians to monitor the state of achievement of trials periodically and encourage or help the participating institutions to collect accurate information in order to prevent loss to follow-up. For example, reminder letters or newsletters may often be sent to the participating physicians. However, if the physicians seem to be reluctant to submit the necessary periodic reports or don't give the information about their patients, clinical research coordinators (CRC) or principal investigators belonging to the coordinating center may ultimately have to visit the institutions. These additional approaches involve considerable time, labor, and cost, if there are many such physicians or the trials were continued over the long-term. For practical clinical trials, it is essential to perform such additional work as efficiently as possible.

In a multi-center trial, the coordinating center mainly contacts the individual physicians or clinical staff. If the coordinating center could identify in advance what kind of physicians don't report their case report forms (CRF) periodically to the coordinating center, it might lead to an important strategy for more efficient conduct of such trials. However, there has not been much attention focused on what factors are associated with physicians' compliance to date. The objective of the study presented here was to identify the physician factors associated with the need for special visits from coordinating center staff or with failure to supply complete information in an investigator-initiated clinical trial.

## Methods

### Subjects and setting

We used data from a large-scale randomized clinical trial known as the Candesartan Antihypertensive Survival Evaluation in Japan (CASE-J). CASE-J is a prospective, multi-center, open-label, investigator-initiated RCT for high-risk hypertensive patients. The objective is to compare the effectiveness of an angiotensin II receptor antagonist (candesartan cilexetil) and of a calcium channel blocker (amlodipine besilate) in terms of the incidence of cardiovascular events [2]. Enrollment began in September 2001 and 4,728 patients had been recruited by December 2002. This is one of only a few large investigator-initiated RCT in Japan and follow-up was to be completed in December 2005. We used 2 years (from January 2003 through December 2004) of patient follow-up data from the CASE-J trial.

For the CASE-J trial, contracts were made between participant physicians and the Evidence Based Medicine Collaborative Research Center (EBM Center) of the Kyoto University Graduate School of Medicine. The follow-up data were collected every 6 months. Nine central CRCs in the EBM center were mainly responsible for coordinating the data collection from participant physicians and for receiving, editing, processing, and storing data generated in the trial using a Web-based remote data entry system or a fax-based system. A list with the dates of patients' examinations and e-mail or fax reminders were periodically sent to the participating physicians from 2 months before the examination until the completed CRF had been received. If the CRF had not been received 2 months or more after the planned reporting date, CRCs reminded the physicians to submit the periodic report. If, in spite of the reminder, the report had still not been submitted, a CRC and an investigator from the EBM center visited the institution in person to encourage and help with filling out the CRF. This constitutes additional work. If no information was received for a year or more, the eventual outcome is likely to be that the patient data are lost to follow-up.

### Categorization of participating physicians

Each periodic report of patients' data was categorized into one of four mutually exclusive groups. The first was the "complete dataset group", comprising periodic reports submitted to the EBM center within the allowable time window. The second was the "delayed group", comprising reports not submitted within the allowable time window, but eventually submitted without the need for a special visit. The third was the "additional assistance group", comprising periodic reports not sent in within the allowable time window, but finally submitted after additional assistance by means of an actual visit to the institution. The fourth was the "incomplete dataset group", where no periodic reports at all were received by the EBM center. Data unobtainable because of patient death were not considered to be part of the "incomplete dataset".

After the categorization of patients' datasets, we categorized the physicians into two groups, "compliers" and "non-compliers". Non-compliers were defined as physicians with 50% or more of the data submitted categorized as "additional assistance group" or "incomplete dataset group". All other physicians were categorized as compliers.

### The questionnaire

Our questionnaire was previously described in the parent study [3-5]. Briefly, the questionnaires were sent to all physicians participating in the CASE-J trial in January 2003, immediately after the patient recruitment period ended in December 2002. For our study, we used the following physician characteristics (obtained at the start of the follow-up): background factors (age, sex, prior participation in a clinical trial, current participation in another study, place of work, and use of Internet or fax for recruitment and follow-up) and four reasons for participating in CASE-J. These four reasons for participating in CASE-J were: 1) the study protocol was simple; 2) a special interest in the use of the Internet for case registration and follow-up study; 3) expectation that it would be easy to persuade patients to participate in this study; and 4) impression that the support system for case registration and follow-up study was well organized.

### Incremental cost for additional work

In addition, since it was unclear how much additional work costs, we examined such costs in the context of CASE-J. We defined the incremental cost for additional tasks as the additional payment for the CRC plus additional cost for travel plus other overheads. Regular payment for the CRC and other recurring costs, including the cost of regular meetings and normal overheads, were defined as routine costs for the clinical trial.

During the follow-up stage, a fee per patient is routinely paid to the physician concerned every time a periodic report is submitted. If a cardiovascular event occurs, an evaluation fee per patient is paid to the Event Evaluation Committee. We excluded these fees from the total follow-up cost in our study, as well as expenditures for maintenance of the Web-based system and telephone or fax charges, which were considered regular expenditures. All additional costs were converted at a rate of 110 Japanese yen to one US dollar.

### Statistical analysis

P values were calculated based on the χ^2 ^test. Odds ratios and corresponding 95% confidence intervals (95% CI) were computed for the various background factors of the physicians and the number of patients they recruited for a comparison of compliers and non-compliers. If the odds ratio was greater than 1.0, it was considered to indicate that the factor might be associated with the non-complier group. If the 95% CI did not include 1.0, we considered the association to be significant. Multiple logistic regression analysis was also performed to adjust for the confounding factors and to evaluate predictive factors of the non-complier group. We defined a non-complier as a physician with 50% or more of the data submitted categorized as "additional assistance group" or "incomplete dataset group". This threshold of 50% was arbitrary to some degree. We therefore changed this threshold from 50% to from 10% to 90% for the sensitivity analysis. The maximum change in odds ratios and P values were indicated. Physicians' background factors and the number of patients they recruited were assumed to be independent variables. SAS for Windows, release 8.02 (SAS Institute Inc., Cary, NC) was used for all analyses.

## Results

### Patients' dataset categorization

Failure to obtain written consent resulted in the exclusion of 23 of the 4,728 patients entered in CASE-J. Datasets for only eight of the remaining 4,705 patients were not submitted at all during the 2-year follow-up at December 2004.

Datasets for 700 (14.9%) of 4,705 patients were categorized as "additional assistance group" or "incomplete dataset group" during the 2-year follow-up. Data collection for 197 of these 700 patients were additionally assisted by CRCs or investigators from the EBM center at least once during the 2-year follow-up, while 168 (3.6%) of the 4,705 patients withdrew their consent and 335 (7.1%) patient reports were not submitted at some time during the 2-year follow-up.

### Physicians' characteristics associated with the additional tasks

The questionnaire was returned by 448 of the 512 (87.5%) physicians, 22 of whom were excluded from this analysis because 13 were anonymous and nine were replaced during the follow-up period. Response proportions for the questionnaire were 86.4% (357 of 413) for the "complier" group and 69.7% (69 of 99) for the "non-complier" group, showing a statistically significant difference (P < 0.001).

Table 1 shows a comparison between the "complier" and "non-complier" groups in terms of physicians' characteristics and the number of patients recruited. The latter was skewed positively for both groups. Four factors were significantly associated with (non-)compliance: Age over 50 (OR = 1.75; 95%CI = 1.01–3.05), prior participation in clinical trials (OR = 0.43; 95%CI = 0.25–0.75), physicians who thought the support system for case registration and follow-up was well organized (OR = 0.46; 95%CI = 0.27–0.78), and the number of patients recruited (OR = 2.31; 95%CI = 1.10–4.85).

**Table 1:** Physicians' factors and respective odds ratios

	Non-Complier (n = 69)	Complier (n = 357)	Odds Ratio (95% CI)
Age			
- mean (SD)	47.1 (7.2)	49.9 (8.4)	
- median (range)	46 (31–67)	49 (31–77)	
			
- <50 (%)	44 (66.7)	188 (53.3)	1.75^a ^(1.01–3.05)
- ≧50 (%)	22 (33.3)	165 (46.7)	-
Sex			
- Male (%)	62 (92.5)	335 (94.6)	0.70 (0.25–1.95)
- Female (%)	5 (7.5)	19 (5.4)	
Prior experience of participating in clinical trial			
- Yes (%)	42 (62.7)	281 (79.6)	0.43^a ^(0.25–0.75)
- No (%)	25 (37.3)	72 (20.4)	-
Current participation in other study			
- Yes (%)	44 (65.7)	245 (69.0)	0.86 (0.49–1.49)
- No (%)	23 (34.3)	110 (31.0)	-
Working site			
- University, national or private hospital (%)	30 (44.8)	133 (37.8)	1.34 (0.79–2.26)
- Own private clinic (%)	37 (55.2)	219 (62.2)	-
Using Internet or FAX for recruitment and follow-up			
- Using both (%)	14 (20.3)	72 (20.2)	0.78 (0.38–1.59)
- Using Internet (%)	28 (40.6)	176 (49.3)	0.64 (0.36–1.14)
- Using FAX (%)	27 (39.1)	109 (30.5)	-
Simplicity of study protocol			
- Agree (%)	36 (53.7)	205 (61.0)	0.74 (0.44–1.26)
- Neutral or Disagree (%)	31 (46.3)	131 (39.0)	-
			
Special interest in the use of Internet for this trial			
- Agree (%)	22 (32.8)	128 (38.7)	0.78 (0.44–1.35)
- Neutral or Disagree (%)	45 (67.2)	203 (61.3)	-
			
Expectation that it would be easy to explain to patients about CASE-J			
- Agree (%)	14 (20.6)	74 (21.9)	0.92 (0.49–1.76)
- Neutral or Disagree (%)	54 (79.4)	264 (78.1)	-
			
Well-organized support system for case registration and follow-up			
- Agree (%)	29 (43.3)	212 (62.5)	0.46^a ^(0.27–0.78)
- Neutral or Disagree (%)	38 (56.7)	127 (37.5)	-
Number of recruiting patients			
- mean	5.9 (10.4)	10.3 (22.8)	
- median (range)	2 (1–65)	4 (1–200)	
			
- <10 (%)	60 (87.0)	265 (74.2)	2.31^a ^(1.10–4.85)
- ≧10 (%)	9 (13.0)	92 (25.8)	-

a: Significantly associated

Multiple logistic regression analysis showed similar results for the various characteristics and factors. Prior participation in clinical trials (OR = 0.40; 95%CI = 0.21–0.74), physicians who thought the support system for case registration and follow-up was well organized (OR = 0.41; 95%CI = 0.22–0.75), and the number of patients recruited (OR = 2.25; 95%CI = 1.01–5.02) were also significantly associated with (non-)compliance. The sensitivity analysis was also done. The results generally tended to be relatively similar even if the threshold was changed. The odds ratios and p-values were changed as follows: prior participation in clinical trials (OR = 0.39–0.55, P = 0.004–0.04), physicians who thought the support system for case registration and follow-up was well organized (OR = 0.41–0.59, P = 0.003–0.03), and the number of patients recruited (OR = 1.75–2.74, P = 0.03–0.12).

### The incremental cost for additional work

In addition, we examined the incremental cost for additional work, which was about US $112,000 (14.4% of the total follow-up routine costs) and remained constant for the 2 years of the follow-up. The average cost for additional tasks per patient amounted to about US $570.

## Discussion

In this study, we investigated identified the physicians' characteristics and factors associated with physician's compliance and the incremental costs involved in additional work in a clinical trial. CASE-J is one of the few studies that as a large-scale RCT in the cardiovascular disease field worked well in Japan. Investigator-initiated clinical trials have recently been attracting great interest and the results are considered more reliable than those of trials conducted by pharmaceutical companies [6]. However, investigator-initiated clinical trials often suffer from insufficient funding. If trial networks are to be well organized, it is important that trials are conducted more efficiently. Our results should therefore be useful for understanding the situation in Japan and in conducting similar investigator-initiated clinical trials in the future.

The results of our multiple logistic regression analysis showed that three factors were significantly associated with non-compliance: prior participation in clinical trials, physicians' opinion that this trial had a well-organized support system for case registration and follow-up, and a large number of patients recruited for the trial. The first and second factors were to be expected, because prior experience can provide the expertise needed for a new clinical trial, and physicians who feel they are adequately supported can submit their periodic report without seeing it as an extra burden. On the other hand, we had expected that a small number of patients would constitute a smaller burden. However, the physicians who enrolled a large number of patients tended to be more compliant. This may indicate that physicians who enroll large number patients are relatively more assertive about participation in the trial and even if they miss a periodic report, they tend to be more careful thereafter. In the sensitivity analysis, the third factor was partly not significant when the threshold was 80 and 90%. However, this may be because these threshold levels were too high and so the number of physicians in the category of ≧10 recruited patients and "complier" was reduced. The tendencies of odds ratio were all similar and so the threshold of 50% might be considered acceptable for this study.

There are some limitations to this study. First, because this study targeted only Japanese subjects, ethnic or cultural differences could not be taken into consideration so it is not clear whether our findings can be generalized internationally. Second, other factors may influence the physician's compliance. If patients don't come to the hospital periodically, don't comply with the protocol treatment, or have difficulty with the patient-physician relationship, the physicians may feel reluctant to submit the necessary periodic reports. Identification of patient factors that influence patient's compliance and development of patient's compliance strategies focused on patients have received considerable attention [7-11]. Recently, the significance of the interface between patients and clinical staff as a potential factor in maintaining patient compliance has also been reported [12]. We couldn't adjust for these factors because we didn't include them in our research, but we may need to do so in future. Third, the results of this study were based solely on the factors associated with physicians' non-compliance, so it also remains unclear whether intervention from the central data coordinating center can lead to a reduction in non-compliance. Newell *et al*.'s [13] review of physician-focused interventions, such as the distribution of reminders and educational materials, found that they had little effect. How to best influence participant physicians should be the basis of further study.

For CASE-J, the incremental cost for special assistance was about 14.4% of the total follow-up cost during the 2-year follow-up of 197 patients (4.2%) out of 4,705. This did not include the cost of maintenance of the Internet-based system and telephone or fax charges, the per-patient fee for the physician's CRF, or the cost per patient charged by the Event Evaluation Committee. It is difficult to assess objectively whether this cost should be considered high or not, because it can depend the situation of each of the participating institutions. However, considering that the percentage of follow-up information that was not submitted at some time during the 2-year follow-up was 7.1%, which is relatively low, the cost might be considered reasonable.

Physicians' non-compliance often leads to loss to follow-up. Investigators therefore always have to be careful to minimize the quantity of incomplete information during follow-up and make every effort to minimize data lost to follow-up during randomized clinical trials. We believe that our findings will be useful for reducing the additional burden associated with incomplete follow-up data and data lost to follow-up when planning future trials. In future, we need to examine how to intervene to reduce possible non-compliance factors.

## Conclusion

We identified the physicians' factors associated with the need for special visits from coordinating center staff or with failure to supply complete information in an investigator-initiated clinical trial. Our results are useful for understanding of the situation in Japan and for conducting similar investigator-initiated clinical trials in the future. In future, we need to examine how to intervene to reduce possible non-compliance factors.

## Competing interests

The author(s) declare that they have no competing interests.

## Authors' contributions

KO has been involved in drafting the manuscript or revising it critically for important intellectual content. SM and RM have been involved in preparing and developing the questionnaire. JS conceived of this study, and participated in its design and coordination and helped to draft the manuscript. All authors read and approved the final manuscript.

**Table 2:** Multiple logistic regression analysis to identify predictors of "Non-Complier group"

	**Odds Ratio (95%CI)**
Age	
- <50	1.38 (0.75–2.55)
- ≧50	-
Sex	
- Male	0.76 (0.25–2.32)
- Female	-
Prior experience of participating in clinical trial	
- Yes	0.40^a ^(0.21–0.74)
- No	-
Current participation in other study	
- Yes	1.02 (0.56–1.87)
- No	-
Working site	
- University, national or private hospital	1.55 (0.86–2.80)
- Own private clinic	-
Using Internet or FAX for recruitment and follow-up	
- Using both	0.59 (0.30–1.15)
- Using Internet	0.59 (0.26–1.33)
- Using FAX	-
Simplicity of study protocol	
- Agree	0.84 (0.44–1.58)
- Neutral or Disagree	-
Special interest in the use of Internet for this trial	
- Agree	1.01 (0.53–1.94)
- Neutral or Disagree	-
	
Expectation that it would be easy to explain to patients about CASE-J	
- Agree	1.44 (0.67–3.11)
- Neutral or Disagree	-
	
Well-organized support system for case registration and follow-up	
- Agree	0.41^a ^(0.22–0.75)
- Neutral or Disagree	-
Number of recruiting patients	
- <10	2.25^a ^(1.01–5.02)
- ≧10	-

a: Significantly associated

## References

[B1] Meinert C (1986). Clinical trials design, conduct, and analysis.

[B2] Fukui T, Rahman M, Hayashi K (2003). Candesartan Antihypertensive Survival Evaluation in Japan (CASE-J) trial of cardiovascular events in high-risk hypertensive patients: rationale, design, and methods. Hypertens Res.

[B3] Rahman M, Morita S, Fukui T, Sakamoto J (2004). Physicians' choice in using Internet and Fax for patient recruitment and follow-up in a randomized controlled trial. Methods Inf Med.

[B4] Rahman M, Morita S, Fukui T, Sakamoto J (2005). Physicians' attitudes towards and reasons for participation in the Candesartan Antihypertensive Survival Evaluation in Japan (CASE-J) Trial. Journal of Epidemiology.

[B5] Rahman M, Morita S, Fukui T, Sakamoto J (2004). Physicians' reasons for not entering their patients in a randomized controlled trial in Japan. Tohoku J Exp Med.

[B6] Donnan GA, Norris J, Hachinski V (2001). From clinical trials to clinical practice: Prevention of Stroke.

[B7] Orr PR, Blackhurst DW, Hawkins BS (1992). Patient and clinic factors predictive of missed visits and inactive status in a multicenter clinical trial. The Macular Photocoagulation Study Group. Control Clin Trials.

[B8] Shumaker SA, Dugan E, Bowen DJ (2000). Enhancing adherence in randomized controlled clinical trials. Control Clin Trials.

[B9] Wilcox S, Shumaker SA, Bowen DJ (2001). Promoting adherence and retention to clinical trials in special populations: a Women's Health Initiative Workshop. Control Clin Trials.

[B10] Janson SL, Alioto ME, Boushey HA, Asthma Clinical Trials Network (2001). Attrition and retention of ethnically diverse subjects in a multicenter randomized controlled research trial. Control Clin Trials.

[B11] Sprague S, Leece P, Bhandari M (2003). Limiting loss to follow-up in a multicenter randomized trial in orthopedic surgery. Control Clin Trials.

[B12] Jackson M, Berman N, Huber M (2003). Research staff turnover and participant adherence in the Women's Health Initiative. Control Clin Trials.

[B13] Newell S, Bowman J, Cockburn J (1999). A critical review of interventions to increase compliance with medicine-taking, obtaining medication refills, and appointintment-keeping in the treatment of cardiovascular disease. Preventive Medicine.

